# BioQuali Cytoscape plugin: analysing the global consistency of regulatory networks

**DOI:** 10.1186/1471-2164-10-244

**Published:** 2009-05-26

**Authors:** Carito Guziolowski, Annabel Bourdé, Francois Moreews, Anne Siegel

**Affiliations:** 1 INRIA, Centre Rennes – Bretagne Atlantique, Symbiose, Campus de Beaulieu, 35042 Rennes, France; 2 INRIA, Centre Rennes – Bretagne Atlantique, GenOuest, Campus de Beaulieu, 35042 Rennes, France; 3 INRA SIGENAE, Campus de Beaulieu, 35042 Rennes, France; 4 CNRS, Université de Rennes 1, IRISA-UMR 6074, Campus de Beaulieu, 35042 Rennes, France

## Abstract

**Background:**

The method most commonly used to analyse regulatory networks is the *in silico *simulation of fluctuations in network components when a network is perturbed. Nevertheless, confronting experimental data with a regulatory network entails many difficulties, such as the incomplete state-of-art of regulatory knowledge, the large-scale of regulatory models, heterogeneity in the available data and the sometimes violated assumption that mRNA expression is correlated to protein activity.

**Results:**

We have developed a plugin for the Cytoscape environment, designed to facilitate automatic reasoning on regulatory networks. The BioQuali plugin enhances user-friendly conversions of regulatory networks (including reference databases) into signed directed graphs. BioQuali performs automatic global reasoning in order to decide which products in the network need to be up or down regulated (active or inactive) to *globally *explain experimental data. It highlights incomplete regions in the network, meaning that gene expression levels do not globally correlate with existing knowledge on regulation carried by the topology of the network.

**Conclusion:**

The BioQuali plugin facilitates *in silico *exploration of large-scale regulatory networks by combining the user-friendly tools of the Cytoscape environment with high-performance automatic reasoning algorithms. As a main feature, the plugin guides further investigation regarding a system by highlighting regions in the network that are not accurately described and merit specific study.

## Background

Various approaches have been taken to analyse regulatory networks in the last few years. One such approach is *in silico *simulation of fluctuations in network components when the network is perturbed [1]. Comparing *in silico *results with experimental outputs may highlight the relevance of certain approaches in our understanding of biological insights. Nonetheless, confronting experimental data with a regulatory network comes up against many difficulties. One of these is the incomplete state-of-art of the regulatory knowledge, recently identified in [2]. Another difficulty is the large scale of regulatory models, which makes the task of collecting kinetic constants and parameters intolerable. Other issues that induce errors in this confrontation are heterogeneity in the available data [3], and the sometimes violated assumption that mRNA expression levels are correlated to protein activity [4]. To deal with these problems, we propose a *global *qualitative analysis, which formalises automatic reasoning in order to compare experimental data with the network behaviour expected from its topology. Our method highlights incomplete regions in a regulatory model and reasons over which products in the network need to be activated or inactivated in order to *globally *explain the experimental data [5-7].

As described in this article, we have enclosed this reasoning in the BioQuali Cytoscape [8] plugin, which allows the user to visualise the outputs of global automatic reasoning on large-scale regulatory networks. These outputs can be inconsistent subgraphs or a list of nodes, whose expression is deduced as increasing or decreasing in order to explain the observed data consistently.

Consistency between regulatory networks and expression data has been studied previously in [3,9]. These studies proposed manual local consistency checks, in some cases by adding new regulation rules. The automation of this idea was recently implemented in a Cytoscape plugin in [10]. With the BioQuali plugin, we go one step further since we propose automating a global consistency reasoning: the effects of influences are carried through the whole network in order to generate a consistency diagnosis that takes account of the entire topology of the network. In comparison with previous approaches, the new functions we provide are: a global analysis, which involves all network connections; no assumption regarding the null transcription factor activity when its mRNA expression change is non-significant; and visualisation of a consistent configuration of the whole network in which it is inferred that the expression of certain nodes fluctuates in a determined way that explains the experimental data provided. To do this, the BioQuali plugin relies on a robust architecture, which makes it effective in terms of speed and maintainability. BioQuali uses statistically significant expression profiles to deduce activity levels of transcription factors (TFs), however, it does not assume that non-significant mRNA changes imply a null activity level of the TF. The BioQuali plugin can help detect post-transcriptional regulations in a model; this capacity relies on the BioQuali inferred fluctuating nodes. This inference, obtained after confronting consistent transcriptional regulations with mRNA changes, reflects new fluctuations related either to mRNA expression or to protein activity levels. Confronting this inference with the sense of variation of mRNA expression may elucidate missing post-transcriptional interactions. Detecting an inconsistent subgraph may also imply the absence of a post-transcriptional regulation. BioQuali cannot automatically predict when a post-transcriptional interaction is missing, but it does provide tools for diagnosing a regulatory network which, combined with biological expertise, may result in adding post-transcriptional effects to complete a network model.

We illustrate this plugin by means of two regulatory models: a small eukaryotic model of the regulation of fatty acid metabolism and the large-scale prokaryotic transcriptional network of *E. coli*. These networks have been compared with small- and large-scale datasets, obtaining in each case globally-explained inconsistencies and predictions that agree with the biological literature.

## Implementation

**Figure 1:**
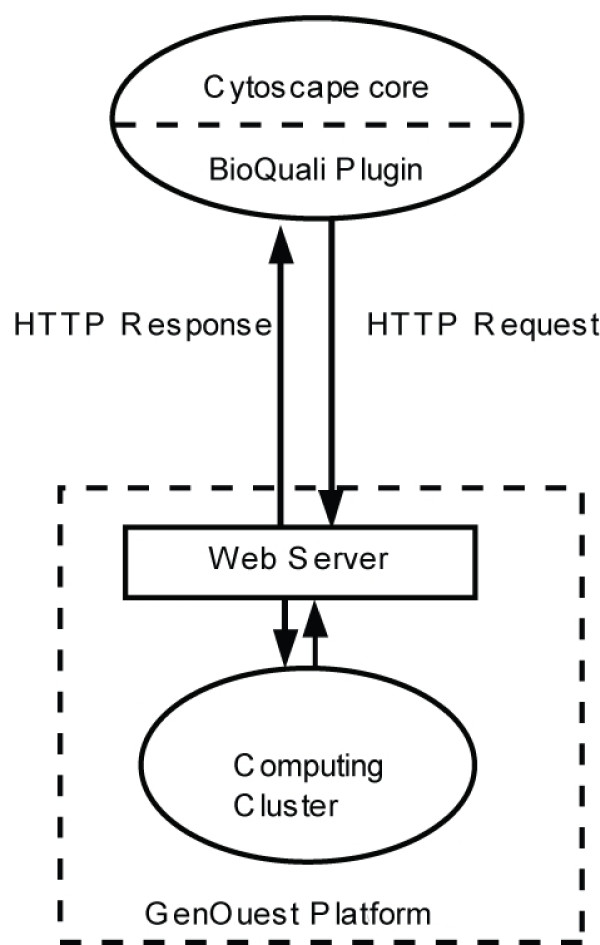
**Plugin architecture**. The BioQuali plugin and the computing cluster communicate over the Internet using HTTP protocol. The plugin first sends an HTTP request containing all the parameters required to launch BioQuali on the GenOuest Web server. Then it periodically sends a request to test the status of the job on the cluster. Once computing is finished, the content of the HTTP response changes and the plugin can retrieve the result and then perform the additional actions to display it correctly in Cytoscape. With this architecture, unauthenticated access to the computing cluster is allowed.

The BioQuali plugin is implemented in Java, based on the Cytoscape API, and using the REST architectural style. By default, the client component uses an unauthenticated HTTP connection to communicate with the GenOuest Web server [11]. This enables fast remote execution of the algorithm underlying the BioQuali plugin on the GenOuest high performance computing facility (see Fig. 1). The GenOuest computing infrastructure consists of 32 AMD Opteron bi-processor nodes (Sun V20Z) with 4 Gb of memory each and a job submission server, SGE, which manages access and computation. Alternatively, the server side component can be downloaded at [12] and installed on any standard PC.

The plugin is available to download from the Cytoscape plugin website [8], under the Plugin/Analysis section. It is packaged as a jar file which must be placed in the Cytoscape plugins directory. It is compiled with the latest Cytoscape API (version 2.6). The plugin can also be installed using the Cytoscape plugin management system, selecting "BioQualiPlugin v.1.1" from the Analysis section. Alternatively, the BioQuali plugin is available via Java Web Start (see [13]). Descriptions and documentation are provided under the "Availability and requirements" section.

## Results

### BioQuali plugin functionalities

#### Inputs

The BioQuali plugin receives two types of input: an experimental dataset and a regulatory network. BioQuali is able to handle large-scale networks, for example, the bacterial transcriptional regulatory networks of *E. coli *[14] and *Corynebacterium *[15]. However, any type of regulatory network is accepted provided that an annotation file with labelled interactions is first imported to Cytoscape. The plugin provides a user-friendly annotation interface for classifying interaction labels as {+, -, &, ?} regulation types. This classification will then be used to perform automatic reasoning on behaviours. The {+, -} types represent positive and negative influences among network products; the '&' is a boolean *AND *among signs (the variation of a product is positive only when all the influences it receives are positive); and the '?' represents interactions with unclassifiable effects (either unknown or context-dependent sign).

Thanks to the annotation functionality, the plugin is compatible with other network import plugins: the user may use regulatory networks obtained using the CoryneRegNet Cytoscape plugin [10] or automatically import the latest update of the RegulonDB database in order to retrieve the *E. coli *transcriptional network [14].

The experimental dataset, resulting from the comparison of gene or protein expression levels between two conditions, can be provided as raw numbers representing the relative gene expression levels. A functionality of the plugin enables the user to classify these observations as up- or down-regulated {+, -} using a chosen threshold. The dataset can also be imported as a Cytoscape node attributes file (.NA) in which certain network products are annotated as '**+**' or '**-**', depending on their expression change.

#### Outputs

**Figure 2:**
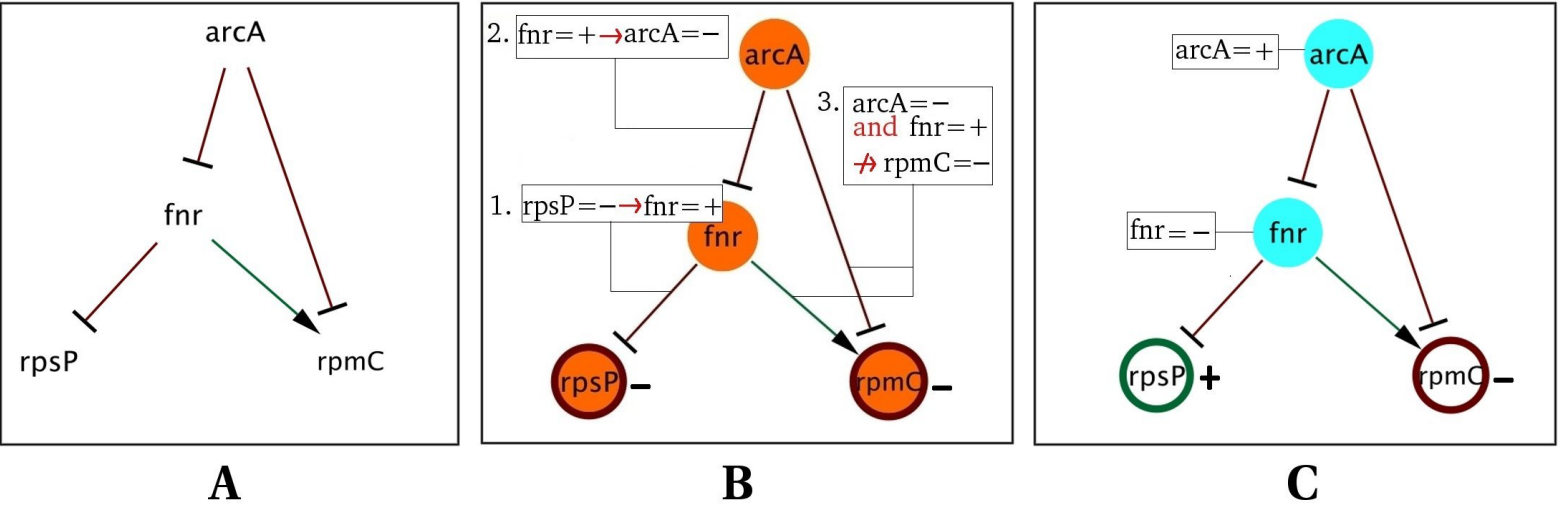
**Automatic reasoning**. Illustrating the consistency criteria. **A**. Signed and oriented regulatory network, arrows ending with '- >' or '-|' represent activations or inhibitions respectively. **B**. Detection of a global inconsistency when *rpsP *and *rpmC *are both observed to be down-regulated. **Step 1**, *rpsP*'s negative change implies that its only inhibitor has to be up-regulated (*fnr *= **+**); **Step 2**, *fnr*'s positive change implies that its only inhibitor has to be down-regulated (*arcA *= **-**); **Step 3**, these deductions cannot explain *rpmC*'s down-regulation, since its activator (*fnr*) is up-regulated and its inhibitor (*arcA*) is down-regulated. **C**. Prediction when *rpsP *is observed to be up-regulated and *rpmC*, down-regulated. *rpsP*'s inhibitor (*fnr*) is fixed to '**-**' to explain the observed value of *rpsP*, in a similar manner, *arcA *is fixed to '**+**'; with this unique configuration we obtain a consistent system where *fnr *= **- **and *arcA *= **+ **are the predictions.

The plugin outputs three types of results after checking for consistency: a list of *local inconsistencies *(LI), *global inconsistencies *(GI) or a *list of predictions*. The first result (LI) is output when the network presents inconsistencies of the form: "A is the only activator of B, A increases but B decreases". The second type of inconsistency (GI), much more difficult to detect, is a global one: it is shown as a subgraph in which the sign of its nodes or edges contradicts the flow of events at certain steps (see the example in the next section and illustrated in Fig. 2B). The plugin automatically retrieves all the subnetworks of a model that are inconsistent with a certain dataset (iteratively, all the interactions of a GI are fixed to '?' and a next GI is computed). For the third type of result, the plugin outputs a list of predictions when a network is consistent with experimental data: it shows fluctuations in the network products inferred as increasing or decreasing in order to consistently explain the experimental data.

### The consistency criteria

An obvious way to study the consistency of a regulatory network with expression data is to consider that the expression changes of some network products have to be explained by the connections existing in the network. In order to increase the expression of a gene either one of its inhibitors must be repressed or one of its activators expressed; if this is not the case the model or data should be examined. This type of reasoning also appears in formalisms based on the causal rule [16], and [3,9]. The domain of validity of this rule covers steady state or gene perturbation expression data with small restrictions, as shown in [5,17]. Dynamical behaviours caused by oscillatory modules, such as negative feed forward loops in the network, will appear to be inconsistent using this criterion; implying that a system with these characteristics does not shift from a stable steady state to another stable steady state.

The central functionality of the BioQuali plugin is to automatically and visually illustrate for the user how to explain experimental observations regarding the regulatory model using the abovementioned reasoning. In Fig. 2, we illustrate the automatic reasoning underlying the BioQuali plugin. Given a known (signed and oriented) regulatory network in which some products are observed, the plugin reasons over the whole network in order to determine its consistency. In Fig. 2A, we describe a small regulatory network. Let us say, for example, that *rpsP *and *rpmC *are observed as down-regulated; we then deduce that, as *fnr *is the only inhibitor of *rpsP*, *fnr *should be up-regulated; if this is the case, following a similar reasoning, we conclude that *arcA *should be down-regulated. To conclude our analysis, we observe *rpmC *as down-regulated, however, its inhibitor is down-regulated and its activator is up-regulated; we should conclude that *rpmC *is up-regulated, yet its observed change tells us the opposite, therefore, we find an inconsistency between model and data (Fig. 2B). Using the same network but changing the observed data, *i.e. rpsP *up-regulated and *rpmC *down-regulated, leads us through another deduction path, in this case it is possible to assign a *unique *{+, -} change value to *fnr *and *arcA *that explains the observed data consistently. This unique deduction is called *prediction *(Fig. 2C).

The problem with global automatic reasoning is that it is computationally difficult to solve when the system is large enough (NP-complete), even for discretised network influences (**+**, **-**, &, **?**) and discretised expression changes (**+**, **-**). The proposed solution, underlying the BioQuali Cytoscape plugin, uses diagram decisions and is described in the Methods section (for details see [6]). Its main relevance is that it handles the consistency of a network with an expression dataset from a global point of view encompassing all the connections between the network components.

### Case Study 1 – Chicken fatty acid synthesis transcriptional network

**Figure 3:**
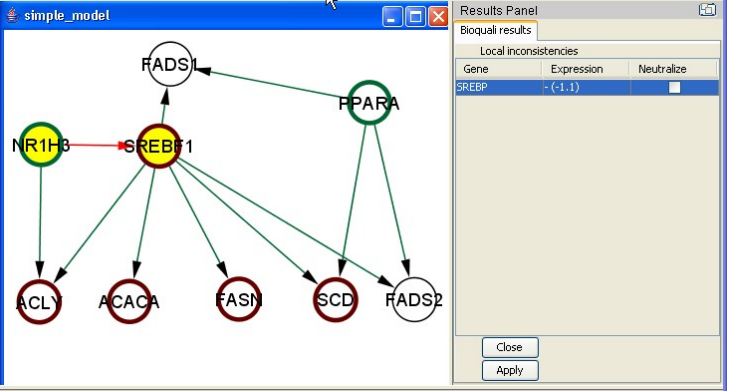
**Fatty acid metabolism, initial model**. Fatty acid synthesis in chicken liver is taken from the literature; only the most important biological entities are represented. After checking the consistency of this model with transcriptomic data detailed in Table 1 (up/down-regulation are represented by nodes with a green/red border respectively), the BioQuali plugin detected a local inconsistency (LI) highlighted in yellow in the graph. The reasoning behind this LI is that SREBF1 is observed in the dataset as down-regulated (-1.1) while its only activator is observed up-regulated, thus, the model should be completed in order to explain SREBF1's observation.

The chicken liver is a major organ, which controls energy metabolism and, especially, fatty acid synthesis. The liver is regulated by complex mechanisms; for this reason, in an initial approach, we used only the most important biological molecules to define a synthetical model. Using the BioQuali plugin, we confronted this model with transcriptomic data obtained from the feeding-to-fasting transition in the chicken liver [18] (Table 1). As an output, we detected the SREBF1 node as a local inconsistency (see Fig. 3): SREBF1 is observed in the dataset as down-regulated, while its only activator is observed as up-regulated, thus, the model must be completed in order to explain SREBF1's observation.

**Table 1:** Transcriptomic results from feeding-to-fasting transition in chicken liver

**Nodes**	**Expression ratio (*log*_2_)**
SREBF1	-1.1
NR1H3	1.2
ACACA	-2.8
FADS1	-0.2
FADS2	-0.6
ACLY	-1.2
FASN	-4.2
PPARA	1.5
SCD	-7.1

*Log*_2 _expression ratios of the biological entities of the fatty acid metabolism network during the feeding-to-fasting transition in chicken liver after 16 hours [18].

In order to correct this inconsistency we completed the model by introducing activated forms of nuclear receptors produced by physical interactions with other molecules. The new nodes were active-PPAR (a complex of RXR and PPARA), active-NR1H3 (a complex of RXR and NR1H3), and active-SREBP (a cleaved form of SREBF1). In the new model, targets of the nuclear receptors PPARA, NR1H3, and SREBF1 became targets of their active forms: active-PPAR is an activator of the transcription of ACACA, FASN, ACLY, SCD1, FADS1, and FADS2 [19,20]; active-NR1H3 is an activator of SREBF1 and FASN, that also indirectly activates ACLY, ACACA, and SCD1 [21].

**Figure 4:**
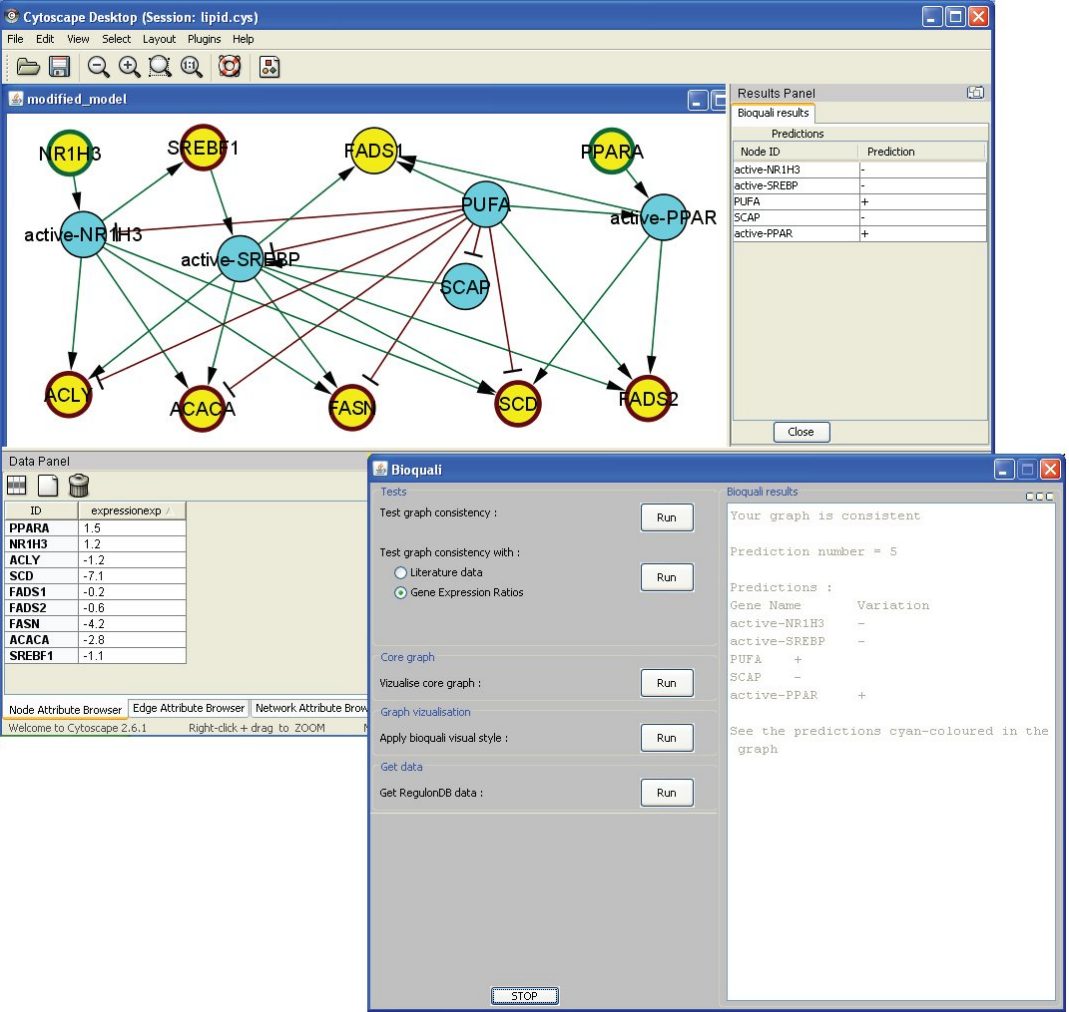
**Fatty acid metabolism, corrected model**. Completed model of fatty acid synthesis in chicken liver, corrected following the inconsistency reported by the BioQuali plugin. The model includes the active forms of PPAR, NR1H3, and SREBP, and a node representing the PUFA (polyunsaturated fatty acid) metabolites. All the new components appear in the literature. This model was consistent with the transcriptome data of Table 1, and the change in expression of PUFA and active-SREBP were predicted. The consistency analysis predictions are listed in the Results Panel to the right.

To comply with this level of detail, we added two nodes standing for control of the activation of the nuclear receptors: SCAP controls the cleavage of SREBF1 while PUFA metabolites (polyunsaturated fatty acids) activate the formation of active-PPAR from PPARA. They also inhibit the formation of active-NR1H3 and active-SREBP [20]. This larger network was globally consistent and output predictions for the nodes without expression values, such as PUFA and active-SREBP (see Fig. 4). These predictions were confirmed by the literature [22]. This analysis suggests that control of nuclear receptors by PUFA metabolites is absolutely necessary to consistently explain the transcriptome experimental data during fasting.

### Case Study 2 – *E. coli *large-scale transcriptional network

**Figure 5:**
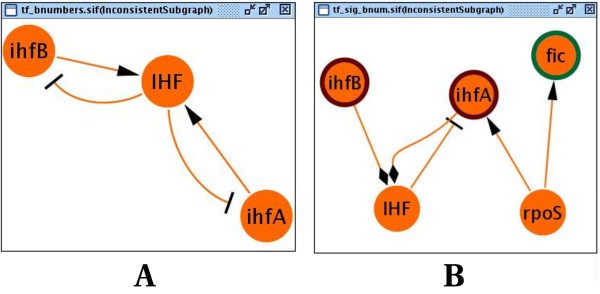
**List of inconsistencies**. List of inconsistencies detected in the *E. coli *transcriptional network. **A**. The inconsistency appears because no possible stable behaviour may be obtained using this network as *ihfA *and *ihfB *genes code for the protein complex IHF, which deregulates the transcription of these genes. **B**. This inconsistency was found after confronting the network with 45 literature-curated expression changes during the exponential-stationary growth shift. The nodes with red and green borders refer to '**+**', and '**-**' observations. The problem appears since no possible explanation exists for the negative shift observed in the *ihfA *expression: *ihfA *is activated by RpoS and repressed by IHF; the change in expression of RpoS was inferred to be positive (because of *fic*), and the change in expression of IHF was inferred to be negative (because of *ihfA *and *ihfB*); consequently, these influences cannot explain the down-regulation of *ihfA*.

The *E. coli *transcriptional regulatory network was obtained from the RegulonDB database [14] on November 2008. It consisted of 3250 TF-gene regulations classified according to three types: activation, repression, and context-dependent effect; we assigned '**+**', '**-**', and '**?**' signs respectively to these three types of regulation. Using the BioQuali plugin, we visualised a region where this network was inconsistent, see Fig. 5A (the complete Cytoscape screenshot appears in the Additional file 1). In order to correct this inconsistency we added the Sigma-gene regulations (all as positive influences), resulting in a network of 5140 regulations. This larger network was globally consistent, meaning that it may entail stable behaviours when experimental data is added to it.

**Figure 6:**
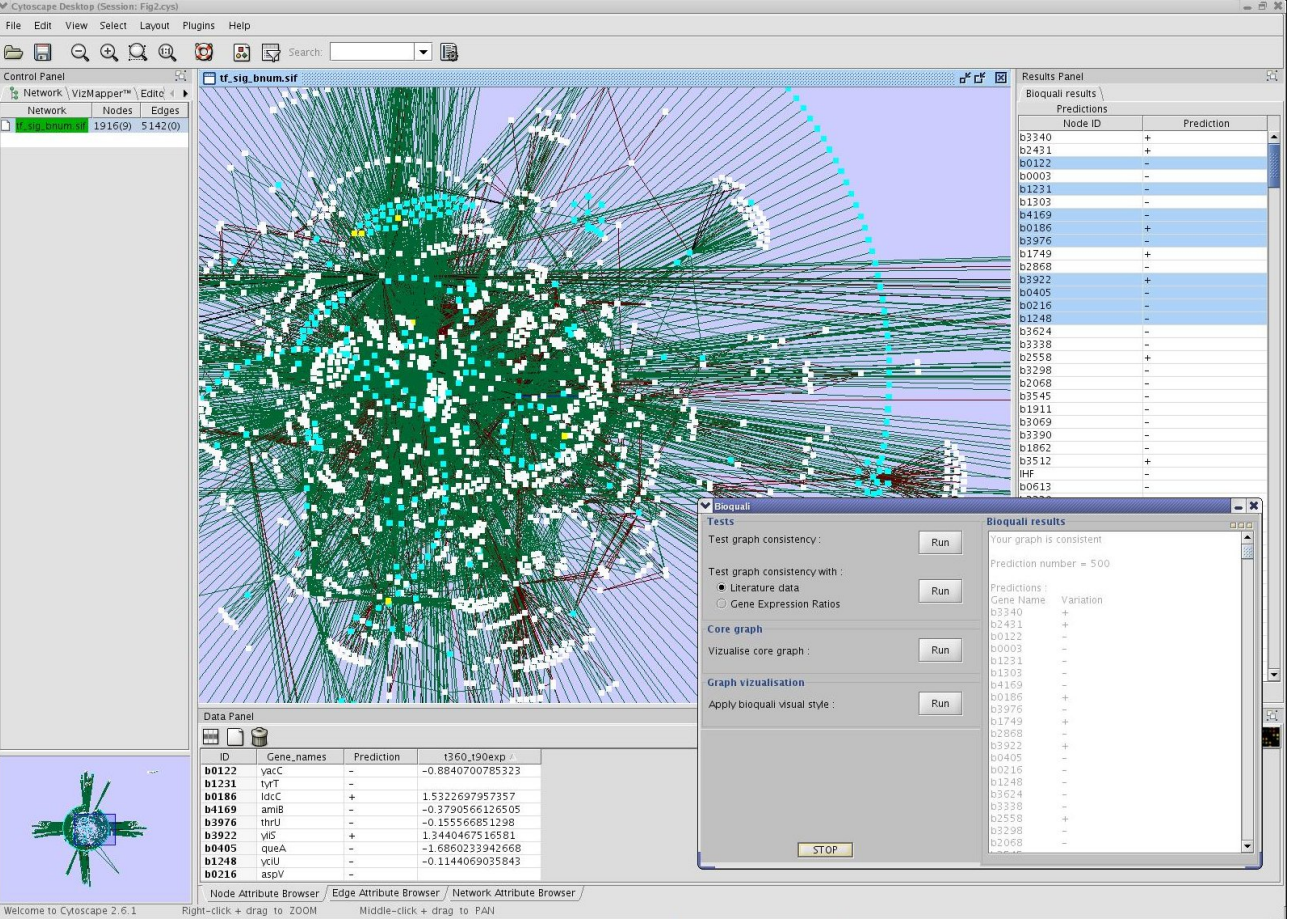
**Visualising the BioQuali predictions**. The predictions shown by the plugin in the *E. coli *network as cyan coloured nodes correspond to network products inferred as {**+**, **-**} in order to explain the 45 gene expression observations initially provided. The plugin lists 498 predicted changes in the Results Panel; it is possible to select and visualise them in detail in the Data Panel and compare them, as shown in this image, to other experimental observations. In the bottom right corner, we see the BioQuali plugin window with all the analysis options that it provides.

**Figure 7:**
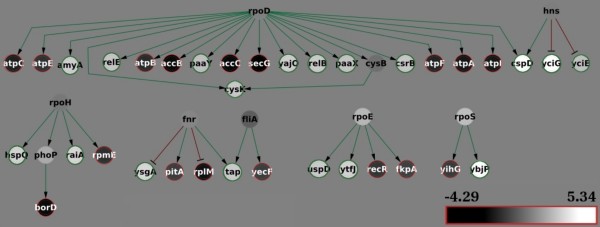
**Multiple global inconsistencies**. Global inconsistencies detected with the BioQuali plugin when checking the consistency of the *E. coli *regulatory network (transcriptional and sigma-gene interactions) with 3-fold significant gene expression changes between the stationary and exponential growth phase [24]. The 16 inconsistent subgraphs output by the plugin were merged into one single subgraph; the expression changes of the network products appear coloured in a black-white scale, the background colour represents no change. The global sigma factor *rpoD *has a non-significant expression change in the dataset. It is shared among many global inconsistent (GI) graphs because its target genes present *significant opposite *changes in the dataset. Since it is not possible to determine a '**+**' or a '**-**' change for *rpoD *that will explain *all *its successors' observations, we detect a GI. The same situation reappears in the successors of *hns*, *rpoH*, and *fnr*. However, the expression change of *fliA*, *rpoE*, and *rpoS *is considerable but not as significant (≤ 3-fold) as that of their successors; reducing the significance threshold to 2-fold will convert these GIs into local inconsistencies.

We compared the globally consistent regulatory network with two experimental datasets: (1) A small literature dataset obtained from RegulonDB in which 45 proteins/genes were carefully verified as increasing ('**+**') or repressing ('**-**') during exponential-stationary growth shift; this was a heterogeneous dataset since the changes were reported at different time-points. (2) A genome-scale dataset obtained by comparing two *E. coli *Affymetrix expression compendia [23]; 4298 {+, -} gene expression changes were obtained by comparing the stationary growth phase with the early-log growth phase in the *E. coli *K12 strain [24]. The small dataset of observations was initially inconsistent with the regulatory model (see Fig. 5B). We corrected it by adding two positive regulations from the sigma factor RpoD to *ihfA *and *ihfB *according to a recent publication on *E. coli *functional regulations [25]. This network correction explained the observed repressed ('**-**') effect of *ihfA*. The consistency of the corrected model with the small dataset reflected 498 positive and negative fluctuations in the network molecules (see Fig. 6). We confronted these predictions with the Affymetrix ratios dataset (4298 changes), observing that the predicted fluctuations agree in a range from 60.3% to 88%, depending on whether they were compared to the full dataset or only to 8-fold significant observations. We also confronted the *E. coli *corrected regulatory model using the genome-scale compendium, obtaining 16 global inconsistencies between the model and the dataset when using 3-fold expression changes (see Fig. 7).

Finally, we compared the BioQuali plugin with the COMA Cytoscape plugin [10]. The two approaches propose different ways of reasoning to determine the consistency of a regulatory network with expression data. The main advantage of the BioQuali plugin, compared with COMA, is its ability to determine a global consistency that takes account of all the influences (direct and indirect) that a node in the network receives. The COMA plugin cannot retrieve any of the multiple global inconsistencies we reported in Fig. 7. We also obtained different results when checking the consistency of the *Corynebacterium glutamicum *regulatory network with the expression dataset provided in [26]. The BioQuali plugin detected that the network model was consistent with the 4-fold gene expression changes and it calculated 123 predictions, whereas the COMA plugin output 5 local inconsistencies using the same data. These inconsistencies were not noticed by BioQuali since it assumes that non-observed nodes, or nodes with small change in expression, may influence other genes by post-transcriptional effects (their mRNA is not correlated with their protein activity). An example of this situation is illustrated in Additional file 2.

In Table 2, we summarise the time performance obtained using the BioQuali plugin to visualise all the results mentioned above on a standard PC, comparing them with those obtained using the COMA plugin. Additionally, in order to measure the prediction performance of the BioQuali plugin, we performed 10-fold cross-validation over 2-fold significant changes of the genome-scale dataset. As a result we obtained that our predictions had a 71.8% of precision and 22% of sensitivity on average. This reflects that our consistency criteria can generate good predictions, even when using imprecise data.

**Table 2:** Consistency check, results and time performance

**Network**	**Interactions**	**Nodes**	**Edges**	**Dataset size**	**BioQuali**		**COMA**	
					
					Results	Time	Results	Time
*E. coli *[14]	TF-gene	1499	3250	-	1 GI	21s.	-	-
*E. coli *[14]	[TF|Sigma]-gene	1916	5140	-	Cons.	21s.	-	-
*E. coli *[14]	[TF|Sigma]-gene	1916	5142	45 [14]	498 Pred.	30s.	10 LI	≤ 1s
*E. coli *[14]	[TF|Sigma]-gene	1916	5142	255 [24]	16 GI	420s.	14 LI	≤ 1s
*Corynebacterium *[15]	Protein-DNA	573	806	65 [26]	123 Pred.	20s.	5 LI	≤ 1s.

This table summarizes the results and time performance (in seconds) obtained after the consistency analysis of the large-scale regulatory networks and experimental datasets used to illustrate the BioQuali plugin. We compared the results obtained with our plugin to those obtained with the COMA plugin using the same data. Using BioQuali, we obtained four types of results: local inconsistencies (LI), global inconsistencies (GI), only network consistency (Cons.), or predictions (Pred.). Using the COMA plugin only local inconsistencies are reported. The difference in the types of results is reflected in the time the plugins took to perform the computation. We tested the time performance with a standard PC.

## Conclusion

The main purpose of the BioQuali plugin is to enhance the visualisation of predictions and inconsistencies in order to facilitate the exploration of large-scale networks with regard to an experimental protocol. In concrete terms, the BioQuali plugin derives automatic deductions from observed behaviours and identifies subgraphs with unexpected behaviour. In our opinion, these global inconsistent subgraphs are the most interesting network areas: either an interaction is missing or a dynamical process occurs that cannot be modelled by intuitive reasoning on variations. In both cases, understanding the mechanisms involved in the inconsistent areas of a network will undoubtedly generate new biological insights. For example, in the fatty acids metabolism model, the inconsistency detected using the BioQuali plugin showed that the initially missing control of nuclear receptors by PUFA metabolites was essential to explain the experimental observation.

Regarding the *E. coli *analysis, we conclude that the RegulonDB database proposes a model of regulations that is highly consistent with the significant fluctuations identified by microarray outputs in the exponential-stationary growth shift. We also illustrate how a small set of variations collected from heterogeneous sources can highlight a new and larger consensus of changes in the network products during this condition. These predicted changes express how the fluctuation in a larger part of the network has to occur in order to explain our observed data.

Further improvements to the BioQuali plugin will consist in adding functionalities related to sign inference in oriented but unsigned regulatory networks [17], incorporating the zero value as a fourth sign of expression change or regulation, adding the possibility of representing an interaction as a boolean function, and building a user-friendly interface for experimental design. Also, we are currently working on setting up a SOAP architecture in our Web server in order to facilitate communications between the BioQuali plugin (or other clients) and the GenOuest Web server.

## Methods

### Approach

Consistency between a regulatory network and experimental data is analysed using a causal rule: *All observed fluctuations in a network product must be explained by an influence received from at least one of its predecessors*. We compare our regulatory network with an experimental dataset obtained by comparing two conditions in the studied organism. Therefore, we classify the network products according to three classes: up-regulated/active products ('**+**'); down-regulated/inactive products ('**-**'); and non-observed products ('X'). We also discretise the network regulations as '+' for positive influences, '-' for negative, and '?' for unknown regulations. Let *g*_*i *_∈ {+, -}, and *t*_*j *_∈ {+, -} be the fluctuation of gene *G*_*i *_and transcription factor *TF*_*j *_respectively during an experimental condition. Let *F*_*ji *_∈ {+, -, ?} be the sign of the regulatory influence coming from *TF*_*j *_targeting *G*_*i*_. For each node *G*_*i *_in the graph that receives *n *influences from different transcription factors, we build the following constraint:



In this way, we map a regulatory network into a system of qualitative constraints. The nodes classed as 'X' represent the variables of the system. Addition and multiplication of signs can be understood as natural addition and multiplication of regulatory influences, that is, if two transcription factors inhibit a gene *G*_*i*_, then *G*_*i *_is down-regulated when both of them are active or expressed. If both transcription factors are down-regulated, then the total influence is an increase in the expression level of gene *G*_*i*_. Natural additions and multiplications allow us to state a *consistency *(≃) between the gene fluctuation and the network topology. The qualitative system will be consistent only if a {+, -} assignment of all the variables of the system exists, in which the constraint + ≃ -, or vice versa, does not appear. The complete tables of the sign operators and the consistency relation (≃) are presented in Additional file 3; to represent more complex regulatory phenomena, we have also added the *AND *sign operator.

Several heuristics were proposed for the resolution of qualitative systems, such as the design of a complete set of rules based on Gaussian elimination [27]. Nevertheless, they cannot be used for solving biological large-scale qualitative systems because such algorithms need backtracking, which increases time-computation. The algorithms that we use in the BioQuali plugin were introduced in [6,28] and they include four main tasks:

• *Reduce the interaction graph in a way that preserves the satisfiability of the system *by iteratively removing the nodes that are not observed and have no successors in the graph. The result of this procedure is a subgraph such that any node is either on a cycle, or has a cycle downstream. This subgraph will be represented as a qualitative system of constraints.

• *Transform the qualitative system in sign algebra into a polynomial function with multiple variables to be solved over the finite field *ℤ/3ℤ. A natural mapping from the sign algebra to this field allows us to interpret the consistency relation ≃ as a simple equality relation in ℤ/3ℤ. In this field, every function appears to be a polynomial function, and the zeros of a system of equation are, equivalently, the zeros of a unique function.

• *Represent the solutions of a polynomial function over *ℤ/3ℤ efficiently. The set of solutions of a polynomial function is represented as a Binary Decision Diagram [29], which is a data structure meant to represent functions on finite domains. Any boolean function can be represented as a rooted, directed acyclic graph that consists of decision nodes (variables) and two terminal nodes (standing for the values 0 and 1): each edge represents an assignment of the variable to the considered value. This tree can be reduced by merging any isomorphic subgraphs and eliminating any node whose two children are isomorphic. With such a representation, there is no more redundancy among subtrees, which dramatically decreases the size of the representation of a polynomial function. The implementation proposed in [6] extends this construction to polynomials with three variables (standing for {+, -, ?} values) and provides very efficient computational time performances.

• *Use the decision diagram structure *to compute solutions, predictions and other properties of a system of qualitative constraints.

## Availability and requirements

Project name: BioQualiPlugin

Project home page:  and 

Documentation and tutorial examples: 

Operating system(s): Platform independent

Programming language: Java 5

License: CECILL version 2 (free open software)

Users should contact Annabel_bourde@yahoo.fr or carito.guziolowski@irisa.fr

## Abbreviations

TFs: Transcription factors; SGE: Sun Grid Engine; LI: local inconsistencies; GI: global inconsistencies.

## Authors' contributions

CG conceived the study. AB developed and implemented the plugin. CG contributed to the full test of the plugin and collected data to analyse the *E. coli *and *Corynebacterium *networks. FC analysed the chicken fatty acid synthesis network. CG wrote the manuscript. AS supervised the project. All authors read, corrected and approved the final manuscript.

## Supplementary Material

Additional file 1 **Complete view of Inconsistencies Detection**. Screenshot of the BioQuali plugin results when an inconsistency is detected. The Results Panel to the right lists the inconsistent edges detected. Click here for file

Additional file 2 **Comparing BioQuali with COMA plugin**. Analysis of the *Corynebacterium *regulatory network using both Cytoscape plugins. Click here for file

Additional file 3 **Sign operators**. Overview of the four operators used in solving the consistency of a qualitative system. Click here for file
