# Supplementary figures and images for: BioQuali Cytoscape plugin: analysing the global consistency of regulatory networks
Source: BMC Genomics. 2009 May 26;10:244. doi: 10.1186/1471-2164-10-244 (PMC2693143; doi:10.1186/1471-2164-10-244)

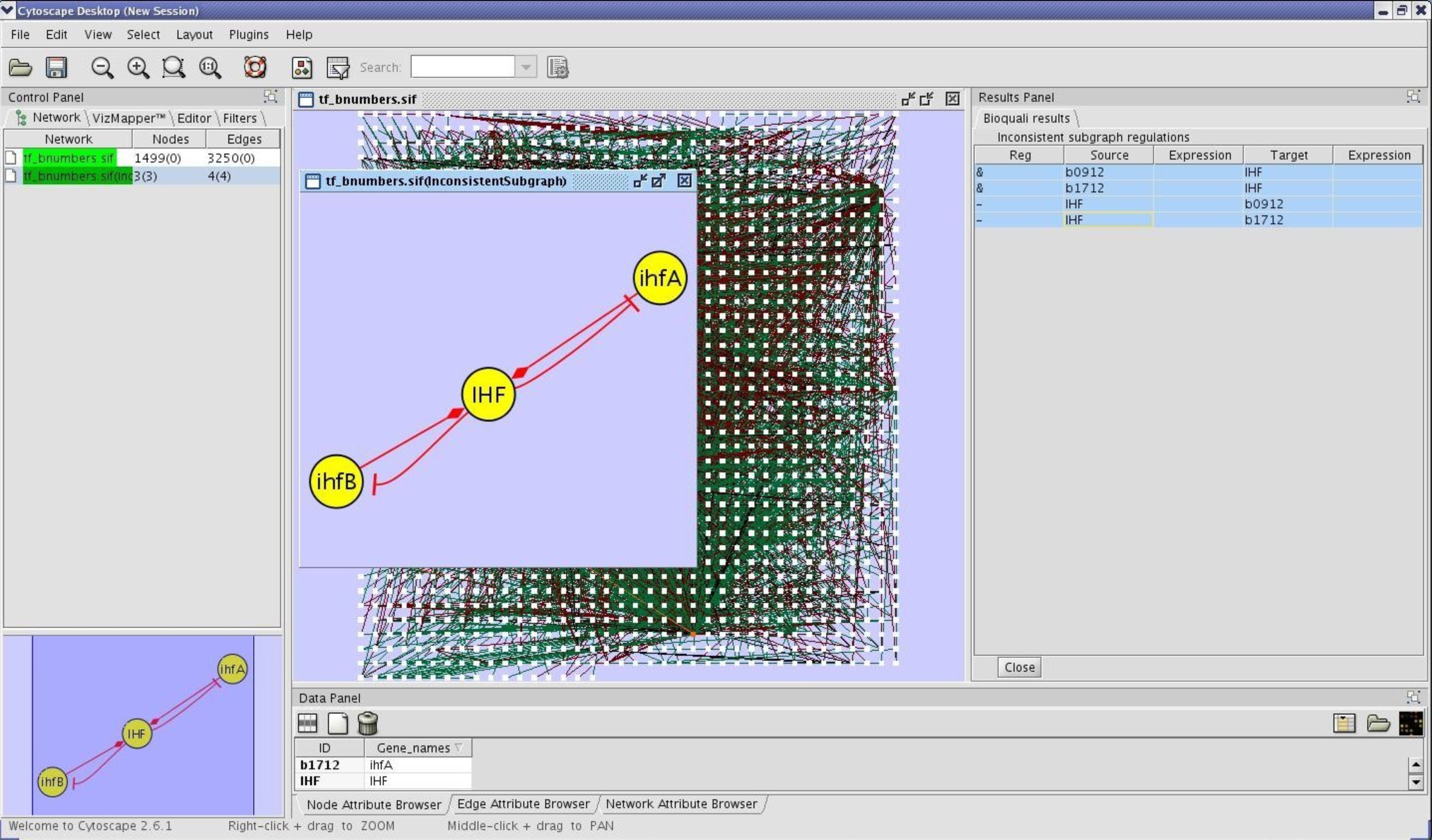

Supplement: Additional file 1 — Complete view of Inconsistencies Detection. Screenshot of the BioQuali plugin results when an inconsistency is detected. The Results Panel to the right lists the inconsistent edges detected. [file 1471-2164-10-244-S1.jpeg]
