# Supplementary material for: BioQuali Cytoscape plugin: analysing the global consistency of regulatory networks
Source: BMC Genomics. 2009 May 26;10:244. doi: 10.1186/1471-2164-10-244 (PMC2693143; doi:10.1186/1471-2164-10-244)
Supplement: Additional file 3 — Sign operators. Overview of the four operators used in solving the consistency of a qualitative system. [file 1471-2164-10-244-S3.pdf]

The $\times$ operator computes one influence by mutliplying the sign of a $TF$ variation by the sign of its influence over a gene. The $+$ and & operators compute the total influence that a gene receives. The $\simeq$ symbol states the consistency answer of each constraint. A qualitative system is consistent if *all* its constraints are consistent.

| $\times$ | $+$ | $-$ | ? |
|---|---|---|---|
| $+$ | $+$ | $-$ | ? |
| $-$ | $-$ | $+$ | ? |
| ? | ? | ? | ? |

| $+$ | $+$ | $-$ | ? |
|---|---|---|---|
| $+$ | $+$ | ? | ? |
| $-$ | ? | $-$ | ? |
| ? | ? | ? | ? |

| & | $+$ | $-$ | ? |
|---|---|---|---|
| $+$ | $+$ | $-$ | ? |
| $-$ | $-$ | $-$ | ? |
| ? | ? | ? | ? |

| $\approx$ | $+$ | $-$ | ? |
|---|---|---|---|
| $+$ | yes | no | yes |
| $-$ | no | yes | yes |
| ? | yes | yes | yes |
