# Supplementary material for: BioQuali Cytoscape plugin: analysing the global consistency of regulatory networks
Source: BMC Genomics. 2009 May 26;10:244. doi: 10.1186/1471-2164-10-244 (PMC2693143; doi:10.1186/1471-2164-10-244)
Supplement: Additional file 2 — Comparing BioQuali with COMA plugin. Analysis of the Corynebacterium regulatory network using both Cytoscape plugins. [file 1471-2164-10-244-S2.pdf]

The consistency of this network with 4-fold significant changes extracted from [1] was analysed using the COMA and BioQuali plugins. The cyan coloured nodes represent the 123 predictions detected with BioQuali. The Results Panel shows the 5 local inconsistencies detected with COMA, one of them is selected and its nodes are coloured yellow in the network. In this selected inconsistency, COMA detects that the interaction from *cg*1585 to *cg*1583 is inconsistent because *cg*1583 is significantly down-regulated while its inhibitor, *cg*1585, is down-regulated as well. BioQuali does not detect this inconsistency, instead, it fixes *cg*3202 (*cg*1583's second inhibitor) as '+' (up-regulated) in order to explain the *cg*1583 down-regulation. Actually, *cg*1583's mRNA may be changing very little in the dataset for multiple reasons, however, this does not mean that its activity level should not be considered.

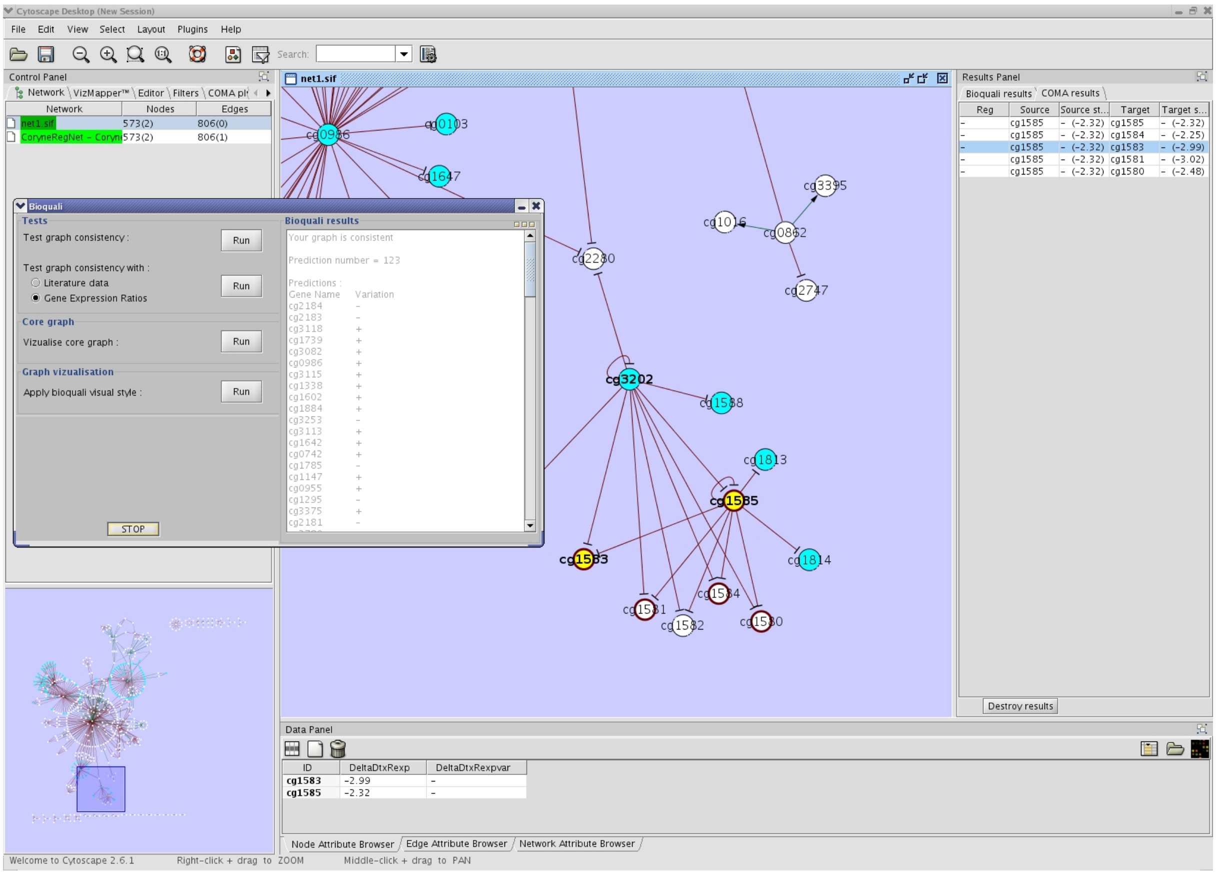

References

[1] Brune I, Werner H, Hüser A, Kalinowski J, Pühler A, Tauch A: **The DtxR protein acting as dual transcriptional regulator directs a global regulatory network involved in iron metabolism of Corynebacterium glutamicum**. *BMC Genomics* 2006, **7**:21.
